# HER2 Status in Gastric and Gastroesophageal Carcinomas: Evaluation of Histopathological Fingings, Paired Resection-Biopsy Specimens, and the Effect of Neoadjuvant Therapy: A Single Center Study*

**DOI:** 10.5152/tjg.2025.24488

**Published:** 2025-01-13

**Authors:** Resmiye Irmak Yuzuguldu, Ozgul Sagol, Mehtat Unlu, Merve Keskinkilic, Serhan Derici, Hale Akpinar, Sulen Sarioglu

**Affiliations:** 1 Department of Medical Pathology, Dokuz Eylül University Faculty of Medicine, İzmir, Türkiye; 2 Department of Molecular Pathology, Dokuz Eylül University Institute of Health Sciences, İzmir, Türkiye; 3 Division of Medical Oncology, Department of Internal Medicine, Dokuz Eylül University Faculty of Medicine, İzmir, Türkiye; 4 Department of General Surgery, Dokuz Eylül University Faculty of Medicine, İzmir, Türkiye; 5 Division of Gastroenterohepatology, Department of Internal Medicine, Dokuz Eylül University Faculty of Medicine, İzmir, Türkiye

**Keywords:** HER2, in situ hybridization, gastric carcinomas

## Abstract

**Background/Aims:**

Accurately determining the human epidermal growth factor receptor 2 (HER2) status is crucial in identifying suitable candidates for targeted therapy in gastric cancer, considering the cost and potential side effects of anti-HER2 treatments. This study aimed to assess HER2 overexpression/amplification prevalence in gastric and gastroesophageal cancer patients, its correlation with clinicopathological characteristics, and the consistency of HER2 status between biopsy and radical specimens.

**Materials and Methods:**

We analyzed data from 667 specimens of 600 gastric/gastroesophageal cancer patients at Dokuz Eylül University Faculty of Medicine from 2012 to 2021. The correlation of HER2 expression and amplification status and clinicopathological parameters were assessed. Furthermore, we compared HER2 status concordance between paired biopsy-radical specimens when both endoscopic and radical materials were present. Additionally, we compared HER2 status before and after treatment in patients receiving neoadjuvant chemotherapy.

**Results:**

In our study, +3 HER2 immunohistochemistry results were more common in gastroesophageal junction tumors (23%). Human epidermal growth factor receptor 2 immunohistochemistry positivity and HER2 gene amplification were found to be significantly more common in males, people over 65 years of age, and intestinal morphology. Overall concordance of HER2 status between radical and biopsy materials was 95.5%.

**Conclusion:**

The high HER2 concordance between the paired biopsy and radical samples holds significant importance in clinical management. This finding is particularly noteworthy since the HER2 assessment can generally be conducted on small/limited biopsy materials in routine practice. Our study is the most extensive series from Türkiye and the Balkans, which will shed light on our country’s data by investigating the relationship of HER2 with clinicopathological parameters in gastric/gastroesophageal carcinomas.

**Main Points**

- Data of 667 specimens obtained from 600 patients with gastric or gastroesophageal cancer were evaluated for HER2 protein expression in biopsy and/or radical specimens.
- Human epidermal growth factor receptor 2 immunohistochemistry positivity and HER2 gene amplification were significantly more common in men, individuals aged over 65 years, and those with intestinal-type morphology. Additionally, a higher frequency was observed in tumors located in the GEJ (23%) compared to other locations.
- High overall concordance (95.5%) was detected between the HER2 status of paired biopsy and radical materials. This finding is important in clinical management, considering that HER2 assessment was conducted solely on small biopsy specimens in many patients.

## Introduction

Gastric cancer (GC), which is the fifth most frequently diagnosed malignancy and the fourth leading cause of cancer-related deaths worldwide, accounted for approximately 800 000 deaths globally in 2020.^1^ Its early and advanced stages are characterized by extensive morphological diversity, leading to the development of numerous classification systems. Laurén^2^ and World Health Organization (WHO)^3^ classifications are the most commonly used systems.Human epidermal growth factor receptor 2 (HER2) is a proto-oncogene and a member of the epidermal growth factor family (EGFR). It is located on chromosome 17 (CR17), which encodes a tyrosine kinase receptor whose phosphorylation initiates many signaling pathways and leads to cell proliferation, differentiation, and apoptosis.^4^ Amplification of the HER2 (also known as ERBB2) oncogene and overexpression of the HER2 protein occurs in approximately 17%-20% of patients with GCs and is more common in intestinal-type GC and cancers located in the proximal stomach or at the gastroesophageal junction (GEJ).^5^^,6^ Human epidermal growth factor receptor 2 is routinely tested as a strongly predictive biomarker for anti-HER2-targeted therapy such as trastuzumab. We aimed to determine the frequency of HER2 overexpression/amplification in gastric/GEJ cancer patients’ biopsy and radical specimens, to demonstrate the correlation of HER2 status with clinical and pathological parameters, concordance of HER2 status between radical and biopsy materials, and to compare HER2 status before and after treatment in patients receiving neoadjuvant chemotherapy (NCT) or systemic cytotoxic therapy.

The present study provides a detailed analysis of the relationship between the HER2 status in gastric and GEJ carcinomas and clinicopathological parameters with patient demographics at both the immunohistochemistry (IHC) and in situ hybridization (ISH) levels in Türkiye. Since our study is a large-scale study, it significantly reflects the cross-section of the Turkish population. Furthermore, our study represents the widest series providing concordance information specific to our country for HER2 status in paired biopsy-radical materials.

## Materials and Methods

Our study included 667 specimens of 600 patients diagnosed with gastric or GEJ cancer, evaluated for HER2 protein expression in biopsy and/or radical specimens at Dokuz Eylül University, Faculty of Medicine, Department of Medical Pathology, between 2012 and 2021. Of these patients, 199 (33.2%) patients were female, and 401 (66.8%) were male. The mean age of all patients was 62.39 ± 12.31, the mean age of females was 61.7 ± 1.02, and the mean age of males was 62.7 ± 0.55.

Ethics committee approval for our study was obtained from Dokuz Eylül University Non-Interventional Research Ethics Committee on 08.02.2018 with the decision number 2018/04-27. Informed consent was not obtained because, for this type of study (retrospective, observational), formal consent was not required.

Data regarding age, sex, tumor localization, histologic type, degree of differentiation, tumor invasion depth, presence of lymphatic, vascular, and perineural invasion, lymph node metastasis status, Lauren and WHO histopathological classifications, and whether they received NCT were obtained from patient files and pathology reports retrospectively.

The presence of HER2 expression was evaluated by IHC, and amplification was evaluated by silver in situ hybridization (SISH) methods. For IHC evaluation, Roche, Cell Marque, Her2/Neu SP3 clone; for SISH evaluations, Roche, Inform HER2 Dual ISH DNA Probe Cocktail was applied. The correlation of HER2 expression and amplification status and clinicopathological parameters were assessed.

According to Lauren’s classification (LC), cases were grouped into 4 categories: intestinal type (IT), diffuse type (DT), mixed type (MT), and not otherwise specified (NOS). Cases with mixed diffuse and intestinal carcinomas, mixed adenoneuroendocrine, and adenosquamous carcinomas were classified as MT. Carcinoma with lymphoid stroma (CWLS) and carcinosarcoma, classified under the title of other histological types in the WHO 2019 classification, were included in the NOS category. In addition, according to the WHO 2019 classification, the cases were grouped as papillary, tubular, mucinous, poorly cohesive, MT carcinoma, and other histological subtypes.

In cases where both endoscopic and radical materials are present, we compared HER2 status between paired biopsy-radical specimens. Additionally, we compared HER2 status before and after treatment in patients receiving NCT.

The data of the in-situ hybridization results of the cases were recorded from the pathology reports. Archive preparations of the cases with missing ISH or IHC data were re-evaluated. Cases diagnosed with gastric/GEJ carcinoma but unknown HER2 status and cases diagnosed as squamous cell carcinoma and pure neuroendocrine carcinoma were excluded from the study.

### Human Epidermal Growth Factor Receptor 2 Evaluation Methods

Human epidermal growth factor receptor 2 IHC and ISH scoring were done based on the Ruschoff/Hofmann method.^7^ They were assigned scores of 0, +1, +2, and +3 according to their immune reactivity. Human epidermal growth factor receptor 2 and CR17 centromere (CEP17) signals were counted in at least 20 non-overlapping nuclei for SISH evaluation at the regions with robust HER2 IHC positivity. Silver in situ hybridization was applied only to samples with IHC scores of +2 and +3. Samples with an HER-2/CEP17 ratio ≥2 by SISH were accepted as positive. Immunohistochemistry scores of 0 and +1 were accepted as negative, and SISH was not performed on these samples.

### Statistical Analysis

Statistical analysis was performed using the Statistical Package for the Social Sciences (IBM SPSS Corp.; Armonk, NY, USA) 24.0 package program. The chi-square test was used for categorical assessments. The median and mean values were stated for non-parametric values. The correlation between HER-2 expression and other parameters was evaluated with the chi-square test. A “*P*” value less than .05 was considered significant.

Whether the variables showed a normal distribution was determined by Shapiro–Wilk analysis. In comparing groups with non-normally distributed variables, the Mann–Whitney *U*-test was used if there were 2 groups, and the Kruskal–Wallis test was used if there were more than 2 groups.

## Results

### Tumors Mostly Exhibit an Intestinal Histological Type and Demonstrate a Male Predominance

A total of 600 patients with gastric/GEJ adenocarcinoma, which were evaluated by HER2 IHC, were included in this study. Of the cases, 331 (55.2%) were radical gastrectomy, 260 (43.3%) were endoscopic biopsy, and 9 (1.5%) were peritoneal biopsy materials. 180 (30%) cases were operated on or biopsied in other centers and referred to our department. Of these cases, 45 (25%) were radical operation materials, 134 (74.4%) were endoscopic biopsies, and 1 (0.6%) was a peritoneal biopsy. One hundred ninety-nine (33.2%) cases were female, and 401 (66.8%) were male. The mean age of all cases was 62.39 ± 12.31, the mean age of females was 61.7 ± 1.02, and the mean age of males was 62.7 ± 0.55. Tumor localization was available in 590 (98%) of 600 cases, of which 199 (33.2%) were antrum/pylorus, 225 (37.5%) were corpus/fundus, 77 (12.8%) were cardia, 47 (7.8%) were located at the GEJ, and 42 (7%) showed diffuse gastric involvement (Table 1).

According to the LC, 216 (36%) of the tumors were DT, 310 (51.7%) were IT, 62 (10.3%) were MT, and 12 (2%) were in the NOS group. According to the WHO 2019 classification, 295 (49.2%) tumors were tubular, 7 (1.2%) were papillary, 8 (1.3%) were mucinous, 216 (36%) were poorly cohesive, 54 (9%) were mixed intestinal and diffuse type carcinomas, 11 (1.8%) were CWLS, 6 (1%) were mixed adenoneuroendocrine carcinomas, 2 (0.3%) were mixed adenosquamous carcinomas, and 1 (0.2%) was categorized as carcinosarcoma (Table 2).

### Significant Correlation Has Been Found Between the Increase in T Stage and the Presence of Distant Metastasis

When the prognostic parameters of 331 radical materials were evaluated, the T and N stages of 25 (7%) cases, all of which were external consultations, could not be determined. Perineural invasion in 24 and lymphatic and vascular invasion of 21 of the same consultation cases could not be appropriately evaluated since not all tumor blocks were delivered to our center. When the T stages of the remaining 306 radical specimens were examined, 29 (9%) were T1a/T1b, 31 (10%) T2, 112 (36%) T3, 113 (36%) T4a, and 21 (6%) T4b; When N stages were evaluated, 70 (22%) were N0, 75 (24%) N1, 56 (18%) N2, 62 (20%) N3a, and 43 (14%) N3b. A statistically significant relationship was found between the pT and pN stages (*P* < .001). The incidence of perineural, vascular, and lymphatic invasion increased as the T stage increased. The relationship between the presence of these 3 parameters and the extent of the T stage was also statistically significant (*P* < .001). The presence of distant metastases increased as the T stage increased, and the relationship between them was found to be significant (*P* = .013) (Table 2).

### The Rate of HER2 Immunohistochemistry +3 Score is More Frequent in Males, Patients Aged 65 and Over, Tumors with Intestinal Type Morphology and Located at the Gastroesophageal Junction

**Figure 1.**
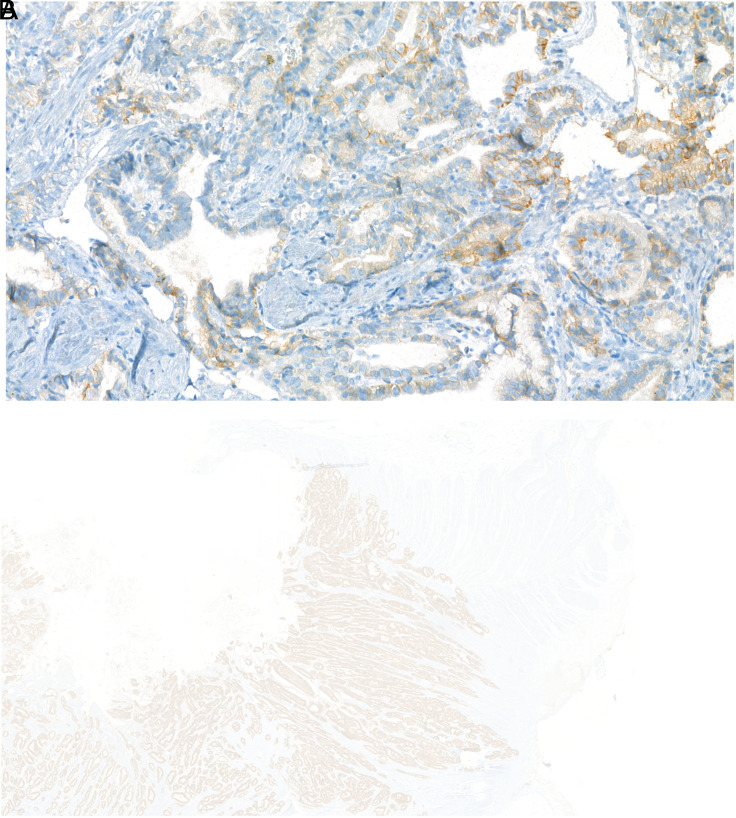
HER2 IHC score of +2 with moderate membranous basolateral staining in a case of intestinal-type adenocarcinoma, ×400 magnification (A), HER2 IHC score of +3 with strong membranous basolateral staining in a case of intestinal-type adenocarcinoma, ×40 magnification (B).

**Figure 2.**
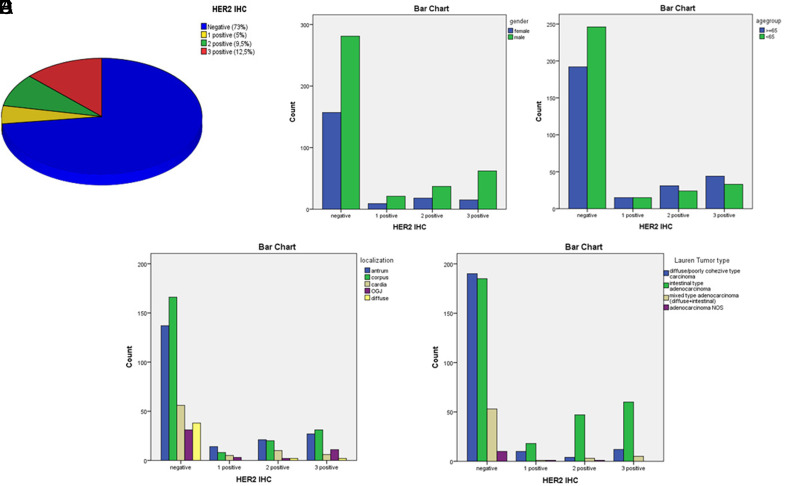
The distribution of cases according to HER2 IHC scores (A), the relationships between gender distribution (B), age groups (C), tumor localization (D), and histological types according to the Lauren classification (E) and HER2 IHC scores.

Human epidermal growth factor receptor 2 protein expression was negative in 438 (73%) patients, +1 in 30 (5%) patients, +2 in 57 (9.5%) patients, and +3 in 75 (12.5%) patients (Figures 1A and B, 2A).

It was observed that the rate of HER2 IHC +3 was statistically significantly higher in males than females (*P* = .034) (Figure 2B). When we divided the cases into 2 groups, 65 years and older and below 65 years of age, it was found that the rate of HER2 IHC +3 was more common in people over 65 years of age, close to the statistical significance limit (*P* = .06) (Figure 2C).

When we compared HER2 IHC positivity rates according to localizations (since the localization of 10 cases could not be reached, 590 cases were evaluated), it was determined that HER2 IHC +3 positivity was higher in GEJ localized tumors compared to other localizations (23.4% of GEJ tumors have HER2 IHC +3). However, no statistical significance was found (*P* = .08) (Figure 2D).

According to the LC, the number of HER2 IHC score +2 and +3 cases in IT adenocarcinomas was 107/310 (34%), while the number of cases in DT carcinomas was 16/216 (7%), and statistically, significantly higher expression was observed in IT adenocarcinomas (*P* < .001) (Figure 2E)

No statistically significant correlation was found between HER2 expression and TNM stage or the presence of perineural, lymphatic, and vascular invasion.

### Amplification of the HER2 Gene Was Detected by In Situ Hybridization in 15.1% of Cases

**Figure 3.**
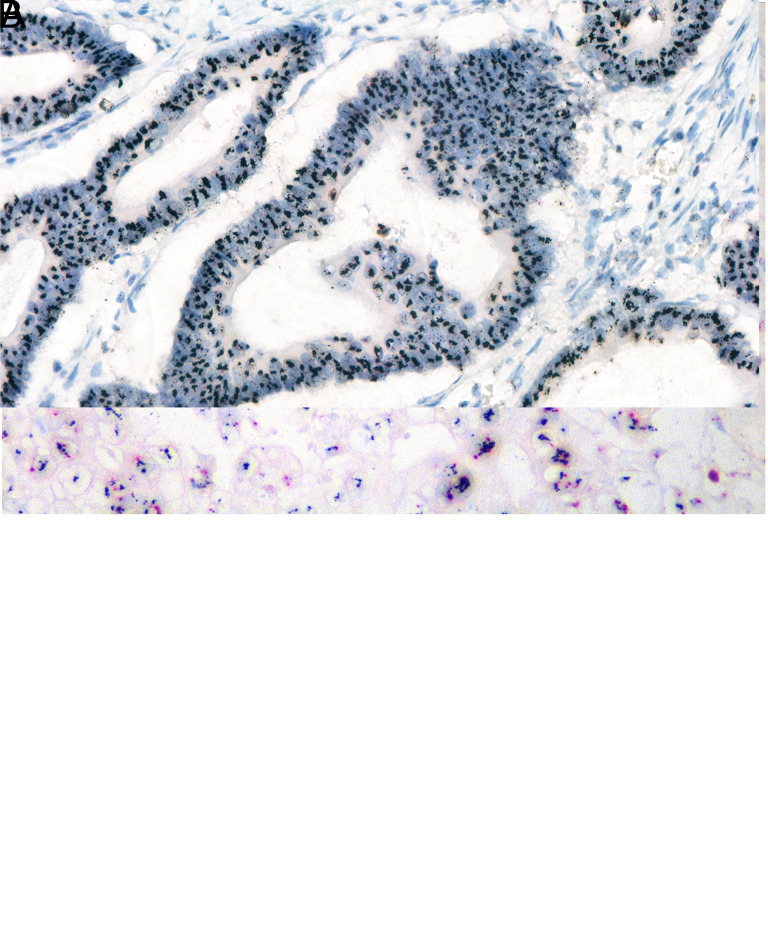
Detection of HER-2 gene amplification using the SISH method. Red: Chromosome 17 gene signals, black: HER2 gene signals (HER2/chromosome 17 ≥ 2), ×200 magnification (A) and ×400 magnification (B).

**Figure 4.**
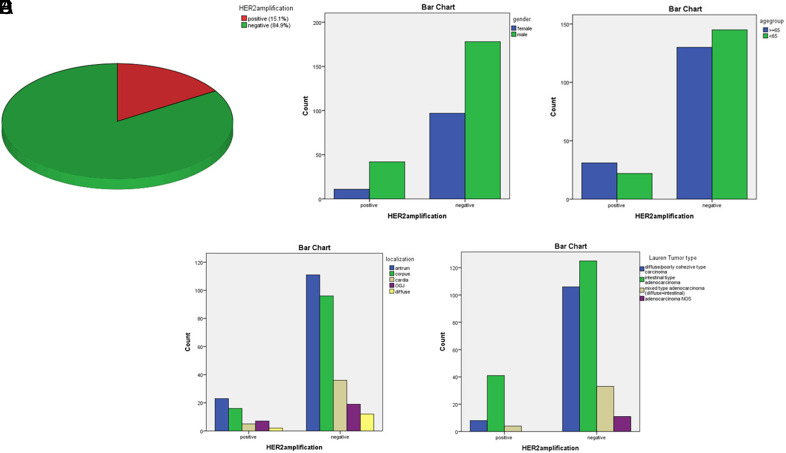
The distribution of cases according to HER2 amplification status (A). The relationships between gender distribution (B), age groups (C), tumor localization (D), and histological types according to the Lauren classification (E) and HER2 amplification status.

Human epidermal growth factor receptor 2 amplification was evaluated in 127 (96%) of a total of 132 cases in whom HER2 IHC +2 and +3 were detected. Amplification was detected in 91 out of 127 patients evaluated for HER2 ISH (Figure 3A and B). Thus, the frequency of HER2 amplification in our series was found to be 15.1% (Figure 4A). Of the cases with HER2 gene amplification, 71 (78%) were male, and 20 (22%) were female, and the presence of HER2 amplification was found to be statistically significantly higher in males (*P* = .013) (Figure 4B). Fifty-seven (62.6%) of the cases with HER2 gene amplification were ≥65 years old, and 34 (37.3%) were <65 years old, and the presence of HER2 gene amplification was statistically significantly higher for those above 65 years of age (*P* = .001) (Figure 4C).

Human epidermal growth factor receptor 2 amplification was detected in 10/46 (21.7%) GEJ cases, 12/77 (15%) cardia cases, 34/222 (15%) corpus/fundus cases, 33/198 (16%) antrum/pylorus cases, and 2/42 (4%) in diffuse tumors involving the whole stomach (Figure 4D). The correlation between HER2 ISH status and clinical data is further presented in Table 3.

It was observed that 79.1% (72/91) of the cases with amplification were IT according to the LC, and the frequency of HER2 amplification was found to be statistically significantly higher in intestinal-type adenocarcinomas (*P* < .000) (Figure 4E). When the HER2 amplification status between subtypes according to the WHO classification is evaluated in tubular adenocarcinomas, the frequency of amplification was found to be statistically significantly higher than poorly cohesive carcinoma, mucinous adenocarcinoma, and CWLS (*P* < .000).

When we evaluated the relationship between tumor histological grade and amplification, it was found that amplification was significantly more common in low-grade tumors (*P* < .000). Perineural invasion in radical materials was statistically significantly more common in tumors without HER2 amplification (*P* = .004). Other histopathological and staging features of the cases with and without HER2 gene amplification are shown in Tables 3 and 4.

### The HER2/CEP17 Ratio and the Frequency of HER2 Gene Amplification Are Higher in Cases with +3 HER2 IHC Score

Human epidermal growth factor receptor 2 amplification was detected in 42% (22/55) of HER2 IHC +2 cases and 95.8% (69/72) of HER2 IHC +3 cases. The detection rate of amplification in HER2 IHC +3 cases was significantly higher than in +2 cases (*P* < .000). When the mean HER2 gene copy number (GCN), CEP17, and HER2/CEP17 ratios were compared in HER2 IHC +2 and +3 cases, it was found that both the mean HER2 GCN and HER2/CEP17 ratio were statistically significantly higher in HER2 IHC +3 cases (*P* < .000). In our series, polysomy was detected in CEP17 in 2 (1.5%) of 127 cases, and the HER2/CEP17 ratio of these cases was >2. The ISH numerical data according to the groups are given in Table 5.

Two of 3 cases with HER2 IHC expression +3, but no amplification detected with SISH, belong to external center consultation blocks. Since no amplification was detected by SISH in these 2 cases, HER2 IHC was repeated, and +3 results were obtained again.

### An 88% Concordance Was Determined Between the HER2 IHC Scores in Paired Biopsy and Radical Materials

In our series, the number of HER2 IHC +3 cases in biopsy materials was 34/269 (12%). In comparison, this rate was found to be 41/331 (12%) in radical surgery specimens, and there was no statistical difference between the 2 groups.

The percentage of cases with HER2 amplification was 14.2% (38/267) in biopsy materials and 16.2% (53/328) in radical surgery specimens, and the difference between the 2 groups was not statistically significant (*P* = .3). In our series, biopsy and radical specimens of 67 cases were available. When the HER2 IHC results of these cases with paired biopsy-radical specimens were evaluated, concordance was found in 54 (80.5%) and discordance in 13 (19.5%).

When we include 5 cases showing discordance as +1/negative in paired specimens, which would not require further ISH examination and would not affect treatment options, in the concordant group as IHC negative, the concordance between radical-biopsy materials of HER2 IHC status was 88%. Human epidermal growth factor receptor 2 IHC expression scores, SISH results, and neoadjuvant treatment status of 13 cases with discordance between paired biopsy-radical materials are shown in Table 6.

### Patients Who Received Neoadjuvant Chemotherapy or Systemic Cytotoxic Therapy Showed An 85% Concordance Between HER2 IHC Scores Before and After Treatment

Of the 600 cases in our series, 77 (12.8%) received NCT or cytotoxic therapy. Of the materials evaluated after NCT or cytotoxic therapy, 73 were radical gastrectomies, and 4 were endoscopic biopsies. Since HER2 IHC was not applied to the pre- and post-treatment materials of 49 patients who received NCT or cytotoxic therapy, a comparison could not be made in these cases.

When the pre- and post-treatment HER2 IHC status of 28 cases was compared, it was found that the IHC scores of 24 (85%) were concordant. In 4 cases, discordance was found between HER2 IHC scores before and after therapy. Characteristics of 4 cases with discordance between HER2 IHC scores before and after therapy are given in Table 7. Accordingly, incompatibility was found to affect the treatment in one of the cases (case number 4). The biopsy material with negative results for IHC belonged to the external center consultation block, ISH was studied in this material, and HER2 amplification was detected.

Of the 28 comparable patients, 22 had negative HER2 IHC scores before and after NCT. Therefore, anti-HER2 agents were not added to the treatment regimens of these patients. In 6 cases, HER2 IHC values before treatment were +3 or HER2 amplification was detected with SISH. Since 3 of these patients were in the metastatic stage, trastuzumab was added to their palliative cytotoxic treatment regimens.

## Discussion

The interpretation of HER2 status is crucial for selecting patients with gastric/GEJ carcinomas who may benefit from HER2-targeted therapy. Considering the expense and side effects of anti-HER2 targeted therapy, further investigation is necessary to select patients who are most likely have high levels of HER2 expression. Clarifying the association between HER2 expression and clinicopathological features offers a convenient way to select patients most likely to have a high level of HER2 expression.

Our study is the most extensive series investigating the relationship between HER2 and clinicopathological parameters in Türkiye and the Balkans. The findings in our study regarding the prevalence of HER2 IHC positivity and amplification of the HER2 gene were significantly more common in males, individuals aged over 65, those with IT morphology, and showed a higher occurrence in proximal tumors, close to the limit of statistical significance, which are consistent with numerous meta-analysis studies.

Human epidermal growth factor receptor 2 status concordance was evaluated in paired radical-biopsy materials in our series, and the overall concordance was found to be high (95.5%). This is important in clinical management because many patients are evaluated only on biopsy material. Considering that the HER2 status detected in the biopsy material determines the treatment options of many patients diagnosed with gastric/GEJ carcinoma and that there is high intratumoral heterogeneity in these tumors,^8^ studies comparing HER2 status in paired biopsy radical specimens have recently started to increase. Moreover, the effect of neoadjuvant or systemic cytotoxic treatment regimens on the expression of HER2 IHC, which has come to the fore with the use of targeted agents in cytotoxic treatment regimens, is one of the issues focused on in the international literature recently.^9^^,^^10^ Therefore, our series investigated HER2 concordance in paired biopsy-radical specimens and the HER2 status of cases treated with neoadjuvant or systemic cytotoxic therapy.

In our series, the rate of HER2 IHC +3 and the presence of HER2 ISH amplification were found to be statistically significantly higher in males (*P* = .034 and *P* = .013, respectively). According to the literature, the incidence of GC in males is twice that in females.^11^ In a meta-analysis study evaluating 15 studies, a statistically significant relationship was found between HER2 positivity and the male gender (*P* < .00001).^12^

We found that HER2 IHC +3 positivity was remarkably higher in GEJ/cardia localized tumors than in other localized tumors (*P* = .08). Studies support that HER2 overexpression is significantly higher in GEJ/cardia localized tumors^5^ or there is no significant correlation between localization and HER2 expression.^13^ This finding might be related to GEJ tumors generally being of IT^14^ and supports that the etiology and pathogenesis of gastroesophageal cancers are different from cancers with a distal location. Unlike distal cancers, the correlation between proximal cancers and gastroesophageal reflux disease has been shown by studies.^15^

Although molecular characteristics (either specific gene alterations or broader molecular subtypes) rather than morphological features might guide future treatment development, LC is still the most commonly used for subgroup analyses in clinical trials. When we compared the relationship between the morphological types according to the WHO 2019 classification and the presence of HER2 amplification in our series of tubular adenocarcinomas, the frequency of detection of amplification was found to be significantly higher than poorly cohesive carcinoma, mucinous adenocarcinoma, and CWLS (*P* < .000). In our study, we found a significant relationship between IT adenocarcinoma and HER2 positivity (figure/table number). This is consistent with a published meta-analysis with a small number of studies investigating the relationship between histological types according to the LC and HER2 overexpression.^12^ In meta-analysis studies, HER2 positivity was detected at a higher rate in well-moderately differentiated tumors (<.0001), similar to our study.^12^ This finding can be explained by the fact that intestinal (Laurén) and tubular type (WHO, 2019) adenocarcinomas are generally well differentiated.

While no correlation was found between perineural invasion and HER2 IHC overexpression, perineural invasion was significantly more common in tumors without HER2 amplification (*P* = .004). In most studies comparing the relationship between HER2 status and clinicopathological parameters, no relationship was found between the TNM stage, the presence of lymphatic, vascular, and perineural invasion, and HER2 amplification.^12^^,^^16^ However, some studies found a statistically significant relationship between the presence of perineural invasion and HER2 IHC overexpression in advanced GCs^17^ and argued that HER2 positivity in early-stage GCs is an independent risk factor for lymph node metastasis.^18^

A multicenter study conducted by Gürbüz et al^19^ involving 552 patients from Türkiye reported that the Lauren classification, venous and neural invasion, and grade were not associated with HER2 status. However, only metastatic gastric carcinomas were included in this study. Therefore, these results are not directly comparable to our study.^19^ In another multicenter study involving partial or total gastrectomy specimens from 212 patients, the incidence of HER2 was determined to be 16.5% (35/212).^20^ In a single-center study involving 135 patients, it was observed that HER-2 positive patients were similar to negative patients concerning age, gender, tumor size, tumor location, tumor T stage, lymph node metastasis, histological type, differentiation, lymphovascular invasion, perinodal, perineural invasion, and stage.^21^

Studies of HER2-positivity rates in GC using immunohistochemistry (IHC) and fluorescence or chromogenic in situ hybridization (FISH/CISH) have shown broad variations, ranging from 6.8% to 34.0 % for IHC,^22^ 7.1% to 42.6% for FISH,^22^ and 12.2% to 24.0% for CISH.^23^ In our study, IHC-related HER2 positivity (+2 and +3 cases) was found at a rate of 22% (132/600), and amplification was detected in 71% (94/132) of these cases. According to ToGA data, IHC +2/ in situ hybridization positive or IHC +3 cases are considered to show overexpression, so the rate of cases with overexpression is 15.6% in our series. Human epidermal growth factor receptor 2 protein overexpression is detected in approximately 17%-22% of primary tumors in GCs, which is consistent with the findings of our study.^5^^,^^6^

According to the ToGA study, among patients whose tumors scored IHC +3, 94.9% (354/373) were FISH-positive, while 54.6% (212/388) were FISH-positive in the IHC +2 group.^5^ Similar rates were obtained in our series, and the detection rate of amplification in HER2 IHC +3 cases was significantly higher than in IHC +2 cases (*P* < .000).

In a study in which 86.4% of the cases were IHC +3, the mean HER2/CEP17 ratio determined by dual-color (dc)-SISH was 6.70, the mean HER2 GCN by dc-SISH was 12.39, and there was a positive and statistically significant correlation between the HER2/CEP17 ratio and HER2 GCN determined by dc-SISH (*P* < .0001).^24^ Similar values were obtained in our series (Table 5). When the mean HER2 GCN, CEP17 GCN, and HER2/CEP17 ratios were compared in HER2 IHC +3 and +2 cases, it was found that both the mean HER2 GCN and HER2/CEP17 ratio were statistically significantly higher in HER2 IHC +3 cases (*P* < .000).

Cases demonstrating ISH CEP17 averaging ≥3.0 were defined as CR17 polysomic.^25^ Approximately 4% of the patients enrolled in the ToGA trial fit into this category.^26^ Chromosome 17 polysomy, detected in 1.5% of our cases where ISH was applied, seemed to have a limited impact on gastric HER2 testing. Research suggests that most cases of ISH “polysomy” result from peri-centromeric amplification and not whole CR17 duplication^26^

The rates of detection HER2 IHC +3 (12% and 12%, respectively) or the presence of amplification (14.2% and 16.2%, respectively) in biopsy and radical materials^27^^,^^28^ correlated with our findings. There are 67 cases in which HER2 IHC evaluation was performed in both biopsy and radical specimen material; 54 (80.5%) HER2 IHC results were concordant between biopsy and radical materials, and 13 (19.5%) were found to be discordant. When we include SISH data along with IHC status, the number of cases with discordance at a level that will change the clinical approach drops to 3 (4%). In this case, the concordance of HER2 status increases to 95.5% in paired biopsy-radical specimens. When we look at the literature data, the IHC concordance rate was 74%^8^ in 54 cases in which only HER2 IHC compatibility was evaluated in paired biopsy radical specimens.

In comparison, the overall HER2 concordance was 91.8%^29^ and 96%^30^ in the series of 61 and 128 cases, respectively, in which HER2 IHC and ISH amplification data were evaluated together. Similar concordance rates were found in other studies,^27^ which investigated the compatibility between paired biopsy-radical specimens. This finding indicates that evaluating HER2 in GEJ and gastric adenocarcinoma biopsy specimens is a valid option for therapeutic management. Since many patients do not have a chance for resection at diagnosis and receive palliative treatment, it is crucial for patient management that biopsy specimens are the appropriate option for evaluating HER2 status.

When we evaluated 13 cases with HER2 IHC discordance among paired biopsy-radical specimens, the biopsy materials of 8 of them were HER2 IHC negative, while varying scores of positivity were detected in the radical materials. Since 4 out of 8 cases with discordance had +2 or +3 positive HER2 IHC results in radical materials, SISH was studied, and HER2 amplification was detected in 2. We observed that the biopsy materials of all cases with HER2 IHC negative and HER2 amplification detected in radical materials were sent to our center as an external center consultation block. This suggests that preanalytical processes may have caused the loss of IHC expression.

Also, some cases of discordance may occur because of intratumoral HER2 heterogeneity in GCs.^31^ Intratumoral heterogeneity refers to both morphological aspects and immunoreactivity of tumor cells to antibodies detecting specific biomarkers, such as HER2.^32^ It can be defined as areas with different HER2 scores within the same tumor. Studies have demonstrated significant HER2 overexpression and amplification heterogeneity in GC, even without morphological heterogeneity.^8^ Gastric cancer has a higher rate of intratumoral heterogeneity of HER2 than breast cancer.^7^ Intratumoral heterogeneity was reported in 69%-74% of GC cases.^33^ Since the rate of intratumoral heterogeneity is extremely high in GC, it is recommended to examine at least 3-4 slides, and ISH analysis should be performed in any case that shows clusters of HER2 +3 positivity, even if they represent less than 5% of tumor cells in studies to detect HER2 positivity.^32^

The primary definition of HER2 positivity in gastric cancer treatment, according to the ToGA trial, relied on IHC 3+ or ISH+. However, documentation on HER2-low gastric cancer, categorized as IHC 2+/ISH-negative or IHC 1+, remains limited, estimated to be within the range of 5.4% to 18.6%. In our study, the HER2-low rate was determined to be 10.5% (63/600). Considering the high heterogeneity displayed in the expression levels of HER2 in gastric/GEJ carcinomas, early investigations involving preclinical studies and preliminary clinical trials have indicated potential antitumor effects of trastuzumab deruxtecan in HER2-low tumors.^34^

Treatment with chemotherapy before surgery in GC patients increases the chance for curative resection and allows an in-vivo response assessment of treatment.^35^ When added to palliative chemotherapy, trastuzumab, an anti-HER2 targeted agent, demonstrated a significant survival benefit in patients with metastatic GC.^36^ Therefore, HER2 is routinely tested as a strongly predictive biomarker for targeted drug therapy. However, whether the integration of HER2-targeting drugs in perioperative chemotherapy may further improve treatment outcomes in patients with HER2-positive is currently being investigated in the “INNOVATION” trial.^37^ In the subgroup of patients with HER2-positive esophagogastric adenocarcinoma, the combination of standard-of-care perioperative FLOT therapy with trastuzumab reported a high pathological complete response (pCR) rate and similarly promising survival data. To further improve the prognosis for this subgroup, the PETRARCA study, a phase II/III study evaluating the efficacy and safety of FLOT in combination with trastuzumab plus pertuzumab, a second anti-HER2 antibody, was planned. However, for patients with metastatic esophagogastric adenocarcinoma, the study did not progressed to phase III based on negative data from the JACOB study published during the conduct of this study. In conclusion, the addition of trastuzumab/pertuzumab to perioperative FLOT significantly improved pCR and nodal negativity rates.^38^

Human epidermal growth factor receptor 2 overexpressing GC has several unique characteristics, including heterogeneity of HER2 expression, loss of dependence on HER2 signaling, and changes in HER2 expression after trastuzumab treatment.^10^ There may be changes in HER2 expression and/or amplification with trastuzumab added to the cytotoxic treatment regimen. The T-ACT investigators observed a rate as high as 69% of HER2 loss between the first and second-line treatment with IHC and FISH testing methods.^11^ Janjigian et al^39^ also reported an analysis of 44 patients with post-trastuzumab tumor tissue samples subjected to a targeted next-generation sequencing panel and observed loss of HER2 amplification in 7 (14%) of the tumors. The effect of cytotoxic therapy on HER2 status was investigated in a series of 25 cases, 6 of which were HER2 positive; it was found that besides trastuzumab added to cytotoxic therapy, cytotoxic therapy alone also reduced HER2 expression.^40^

In our series, in 28 cases whose HER2 IHC scores were comparable before and after NCT or cytotoxic therapy, the concordance rate was found to be 85% (24/28 cases had concordance). The discordance identified in 3 out of 4 cases was insignificant to alter the treatment management. Among these 28 cases, 6 of them showed HER2 IHC +3 or detected amplification. Three out of these 6 patients were administered trastuzumab in addition to cytotoxic chemotherapy, and the remaining 3 patients received only cytotoxic chemotherapy. Consequently, no alterations were observed in the HER2 IHC status of these 6 cases before and after the treatment.

Considering that the imaging and laparoscopic techniques used in staging GCs have developed and become widespread, the probability of detecting advanced-stage disease increases, and palliative treatments will be applied more frequently. For these reasons, the effect of treatment regimens, in which both cytotoxic agents alone and cytotoxic plus anti-HER2 agents are used together, on HER2 protein expression and gene amplification in gastric and GEJ carcinomas should be investigated in more extensive case series in our country and in the world.

While this study contributes valuable insights into the subject matter, it is important to acknowledge its limitations. A potential limitation of the current study is its retrospective methodology from a single institution’s experience. However, the strengths of this research lie in its substantial number of cases, which will significantly contribute as a crucial resource regarding the HER2 status in gastric and GEJ carcinomas in Türkiye. Furthermore, our study represents the most extensive series comparing HER2 IHC status in biopsy-radical materials.

In conclusion, we demonstrated that the rate of HER2 overexpression was 15.6%, and HER2 amplification was 15.1% in GC and GEJCs, consistent with the results in the literature. Human epidermal growth factor receptor 2 IHC positivity and HER2 gene amplification were found to be significantly more common in males, people over 65 years of age, and in tumors with intestinal and low-grade morphology. Additionally, it has been determined that within tumors localized at the GEJ, the incidence of HER2 IHC 3+ positivity was significantly higher (23.4%), approaching the threshold of statistical significance (*P* = .08) when compared to other locations. No statistically significant correlation was found between HER2 expression and TNM stage or the presence of perineural, lymphatic, and vascular invasion. The detection rate of amplification in HER2 IHC +3 cases was notably higher compared to +2 cases. Moreover, statistical analysis revealed that both the average HER2 GCN and the HER2/CEP17 ratio were significantly elevated in HER2 IHC +3 cases. Human epidermal growth factor receptor 2 status concordance was evaluated in paired radical-biopsy materials within our series, revealing a notably high overall concordance rate of 95.5%. This finding indicates that assessing HER2 in biopsy specimens of GEJ and gastric carcinomas is a viable option for guiding therapeutic management decisions.

## Figures and Tables

**Table 1.** HER2 IHC Expression Status and Clinicopathological Parameters

Clinicopathological Parameters	HER2 IHC +3 (%)	HER2 IHC +2 (%)	HER2 IHC +1 (%)	HER2 IHC Negative (%)	*P*
Gender Female Male	14 (18.7) 61 (81.3)	19 (33.3) 38 (66.7)	9 (30) 21(70)	157 (35.8) 281 (64.2)	.034
Tumor localization Antrum/pylorus Corpus/fundus Cardia GEJ Diffuse	26 (13.1) 30 (13.3) 6 (7.8) 11 (23.4) 2 (4.8)	22 (11.1) 21 (9.3) 10 (13) 2 (4.3) 2 (4.8)	14 (7) 8 (3.6) 5 (6.5) 3 (6.4) 0	137 (68.8) 166 (73.8) 56 (72.7) 31 (66) 38 (90.5)	.08
Age ≥65 <65	44 (58.7) 31 (41.3)	31 (54.) 26 (45.6)	15 (50) 15 (50)	192 (43.8) 246 (56.2)	.06

GEJ, gastroesophageal junction; HER2, human epidermal growth factor receptor 2.

**Table 2.** HER2 IHC Expression Status and Histopathological Parameters

Histopathological Parameters	HER2 IHC +3 (%)	HER2 IHC +2 (%)	HER2 IHC +1 (%)	HER2 IHC Negative (%)	*P*
Lauren classification Intestinal type Diffuse type Mixed type NOS	59 (78.7) 11 (14.7) 5 (6.7) 0	48 (84.2) 5 (8.8) 3 (5.3) 1 (1.8)	18 (60) 10 (33.3) 1 (3.3) 1 (3.3)	185 (42.2) 190 (43.4) 53 (12.1) 10 (2.3)	<.001
WHO classification (2019) Tubular adenocarcinoma Papillary adenocarcinoma Mucinous adenocarcinoma Poorly cohesive carcinoma Mixed type Other subtypes *Carcinoma with lymphoid stroma* *Adenosquamous* *Adenoneuroendocrine* *Carcinosarcoma*	56 (74.7) 2 (2.7) 1 (1.3) 11 (14.7) 4 (5.3) 1 (3.3) *0* *0* *1 (3)* *0*	45 (79) 2 (3.5) 1 (1.8) 5 (8.8) 2 (3.5) 2 (3.5) *1 (1.8)* *0* *1 (1.8)* *0*	17 (56.7) 1 (3.3) 0 10 (33.3) 1 (3.3) 1 (3.3) *0* *0* *0* *1 (3.3)*	177 (40.4) 2 (0.5) 6 (1.4) 190 (43.4) 47 (10.7) 16 (3.7) *10 (2.3)* *2 (0.5)* *4 (0.9)* *0*	–
T stage* T1a/T1b T2 T3 T4a T4b	2 (5.1) 5 (12.8) 19 (48.7) 11 (28.2) 2 (5.1)	1 (2.6) 5 (13.2) 18 (47.4) 11(28.9) 3 (7.9)	2 (10.5) 1 (5.3) 8 (42.1) 7 (36.8) 1 (5.3)	24 (11.4) 20 (9.5) 67 (31.9) 84 (40) 15 (7.1)	*P* = .514
N stage* N0 N1 N2 N3a N3b	9 (23.1) 16 (41) 5 (12.8) 5 (12.8) 4 (10.3)	10 (26.3) 12 (31.6) 6 (15.8) 6 (15.8) 4 (10.5)	2 (10.5) 6 (31.6) 3 (1.,8) 6 (31.6) 2 (10.5)	47 (22.4) 43 (20.5) 42 (20) 45 (21.4) 33 (15.7)	*P* = .34
M stage M1 MX	9 (23.1) 30 (76.9)	8 (21.1) 30 (78.9)	3 (15.8) 16 (84.2)	40 (19) 170 (81)	*P* = .904
Perineural invasion* Present Absent	17 (43.6) 22 (56.4)	21 (55.3) 17 (44.7)	12 (63.2) 7 (36.8)	124 (59) 86 (41)	*P* = .178
Vascular invasion* Present Absent	19 (48.7) 20 (51.3)	23 (60.5) 15 (39.5)	9 (47.4) 10 (52.6)	121 (57.6) 89 (42.4)	*P* = .583
Lymphatic invasion* Present Absent	24 (61.5) 15 (38.5)	29 (76.3) 9 (23.7)	11 (57.9) 8 (42.1)	156 (74.3) 54 (25.7)	*P* = .181

*The data of these parameters were evaluated in radical specimens.

HER2, human epidermal growth factor receptor 2; IHC, immunohistochemistry; NOS, not otherwise specified; WHO, World Health Organization.

**Table 3.** Relationship Between HER2 ISH Status and Clinicopathological Data

Clinicopathological Features	HER2 ISH (+) (%)	HER2 ISH (−) and IHC (+1/−) (%)	*P*
Gender Female Male	20 (22) 71 (78)	178 (33.3) 326 (66.7)	.013
Tumor localization Antrum/pylorus Corpus/fundus Cardia GEJ Diffuse	33 (16.7) 34 (15.3) 12 (15.6) 10 (21.7)* 2 (4.8)	165 (83.3) 188 (84.7) 65 (84.4) 36 (4.3) 40 (95.2)	.26
Age ≥65 <65	57 (62.6%) 34 (37.4%)	224 (54.4) 280 (55.6)	.001

GEJ, gastroesophageal junction; HER2, human epidermal growth factor receptor 2; ISH, Immunohistochemistry.

**Table 4.** Relationship Between HER2 ISH Status and Histopathological Parameters

Histopathological Parameters	HER2 ISH (+) (%)	HER2 ISH (−) and IHC (+1/−) (%)	*P*
Lauren classification Intestinal type Diffuse type Mixed type NOS	72 (79.1) 12 (13.2) 7 (7.7) 0	234 (46.4) 203 (79.1) 55 (10.9) 12 (2.4)	<.000
WHO classification (2019) Tubular adenocarcinoma Papillary adenocarcinoma Mucinous adenocarcinoma Poorly cohesive carcinoma Mixed type Other subtypes *Carcinoma with lymphoid stroma* *Adenosquamous* *Adenoneuroendocrine* *Carcinosarcoma*	69 (75.8) 2 (2.2) 0 13 (14.3) 5 (5.5) 2 (2.2) *0* *0* *2 (2.2)* *0*	223 (44.2) 5 (1) 7 (1.,4) 202 (40.1) 49 (9.7) 18 (3.6) *11 (2.2)* *2 (0.4)* *4 (0.8)* *1 (0.2)*	<.000^#^
Grade Grade 1 Grade 2 Grade 3	41 (45.1) 25 (27.5) 25 (27.5)	96 (19) 102 (20.2) 306 (60.7)	*<*.000
T stage* T1a/T1b T2 T3 T4a T4b	2 (4.1) 6 (12.2) 24 (49) 13 (26.5) 4 (8.2)	26 (10.2) 25 (9.8) 87 (34.3) 99 (39) 17 (6.7)	*P* = .18
N stage* N0 N1 N2 N3a N3b	11 (22.4) 18 (36.7) 8 (16.3) 6 (12.2) 6 (12.2)	56 (22) 58 (22.8) 48 (18.9) 55 (21.7) 37 (14.6)	*P* = .26
M stage M1 MX	10 (16.9) 49 (16)	49 (83.1) 205 (84)	*P* = .85
Perineural invasion* Present Absent	27 (55.1) 22 (44.9)	148 (58.3) 106 (41.7)	*P* = .004
Vascular invasion* Present Absent	27 (55.1) 22 (44.9)	144 (56.7) 110 (43.3)	*P* = .83
Lymphatic invasion* Present Absent	33 (67.3) 16 (32.7)	186 (73.2) 68 (26.8)	*P* = .4

^#^The explanation is in the text.

*The data of these parameters were evaluated in radical specimens.

HER2, human epidermal growth factor receptor 2; IHC, immunohistochemistry; NOS, not otherwise specified; WHO, World Health Organization.

**Table 5.** ISH Data of Cases with HER2 IHC Score +2 and +3

HER2 IHC Score	The Average Number of Chromosomes 17	The Average Number of HER2 Gene	Average of HER2/Chromosome 17 Ratios
+2 cases	2.12 ± 0.8	4.35 ± 2.41	2.13 ± 1.2
+3 cases	1.83 ± 0.3	12.53 ± 6.1	7.24 ± 3.7
*P*	.09	<.000	<.000

HER2, human epidermal growth factor receptor 2; IHC, immunohistochemistry.

**Table 6.** Characteristics of Cases with Discordant HER2 IHC Scores in Paired Biopsy-Radical Materials

Case Number	Biopsy HER2 IHC Status	Radical HER2 IHC Status	SISH Result	Neoadjuvant Therapy
1	+1	+2	1.56	Absent
2	+1	Negative	Not applied	Absent
3	+1	Negative	Not applied	Absent
4	+2	+3	2.2	Absent
5	+2	+3	2.22	Present
6*	Negative**	+3	3.75	Present
7*	Negative	+2	2.9	Absent
8*	Negative	+2	1.22	Absent
9	Negative	+1	Not applied	Present
10	Negative	+2	0.37	Present
11	Negative	+1	Not applied	Absent
12	Negative	+1	Not applied	Absent
13*	Negative	+2	2.22	Absent

*The blocks of biopsy materials show the cases submitted to our center as consultation from external centers.

**Following the IHC positive/SISH amplified results of the radical operation specimen, SISH was studied in the biopsy material of this material, and amplification was detected.

HER2, human epidermal growth factor receptor 2; IHC, immunohistochemistry; SISH, silver in situ hybridization.

**Table 7.** Characteristics of Cases with Discordance in HER2 IHC Results Before and After NCT or Cytotoxic Therapy

Case Number	HER2 IHC Status Before Treatment	HER2 IHC Status After Treatment	ISH Status
1	Negative*	+1	Not applied
2	Negative**	+2	No amplification detected**
3	+2	+3	Amplification detected***
4	Negative****	+3	Amplification detected

*It was studied from the IHC consultation block before treatment.

**ISH amplification was evaluated after treatment. The block that IHC is studied before the treatment is the consultation block.

***ISH amplification was positive and evaluated both before and after treatment.

***The biopsy material of the case is the consultation block. Although HER2 was negative in the IHC biopsy material, ISH was also studied, and amplification was detected in the biopsy material.

HER2, human epidermal growth factor receptor 2; IHC, immunohistochemistry; ISH, in situ hybridization.

## Data Availability

The data that support the findings of this study are available from the corresponding author upon reasonable request.
